# Revelation of Proteomic Indicators for Colorectal Cancer in Initial Stages of Development

**DOI:** 10.3390/molecules25030619

**Published:** 2020-01-31

**Authors:** Arthur T. Kopylov, Alexander A. Stepanov, Kristina A. Malsagova, Deepesh Soni, Nikolay E. Kushlinsky, Dmitry V. Enikeev, Natalia V. Potoldykova, Andrey V. Lisitsa, Anna L. Kaysheva

**Affiliations:** 1 V.N. Orekhovich Institute of Biomedical Chemistry, 119121 Moscow, Russia; a.t.kopylov@gmail.com (A.T.K.); aleks.a.stepanov@gmail.com (A.A.S.); kristina.malsagova86@gmail.com (K.A.M.); deepesh.soni@mail.ru (D.S.); 2 N.N. Blokhin Russian Cancer Research Center, 115478 Moscow, Russia; f17-1086@yandex.ru; 3 Institute of Urology and Reproductive Health (Sechenov University), 119992 Moscow, Russia; enikeev_dv@mail.ru (D.V.E.); potoldykovanv@gmail.com (N.V.P.); 4 East China University of Technology, Nanchang 330013, China; 13576998241@mail.ru

**Keywords:** colorectal cancer, protein pattern, digital medicine, ultra-high resolution mass spectrometry, post-translational modifications, omics, postgenomic data

## Abstract

**Background:** Colorectal cancer (CRC) at a current clinical level is still hardly diagnosed, especially with regard to nascent tumors, which are typically asymptotic. Searching for reliable biomarkers of early diagnosis is an extremely essential task. Identification of specific post-translational modifications (PTM) may also significantly improve net benefits and tailor the process of CRC recognition. We examined depleted plasma samples obtained from 41 healthy volunteers and 28 patients with CRC at different stages to conduct comparative proteome-scaled analysis. The main goal of the study was to establish a constellation of protein markers in combination with their PTMs and semi-quantitative ratios that may support and realize the distinction of CRC until the disease has a poor clinical manifestation. **Results:** Proteomic analysis revealed 119 and 166 proteins for patients in stages I–II and III–IV, correspondingly. Plenty of proteins (44 proteins) reflected conditions of the immune response, lipid metabolism, and response to stress, but only a small portion of them were significant (*p* < 0.01) for distinguishing stages I–II of CRC. Among them, some cytokines (Clusterin (CLU), C4b-binding protein (C4BP), and CD59 glycoprotein (CD59), etc.) were the most prominent and the lectin pathway was specifically enhanced in patients with CRC. Significant alterations in Inter-alpha-trypsin inhibitor heavy chains (ITIH1, ITIH2, ITIH3, and ITIH4) levels were also observed due to their implication in tumor growth and the malignancy process. Other markers (Alpha-1-acid glycoprotein 2 (ORM2), Alpha-1B-glycoprotein (A1BG), Haptoglobin (HP), and Leucine-rich alpha-2-glycoprotein (LRG1), etc.) were found to create an ambiguous core involved in cancer development but also to exactly promote tumor progression in the early stages. Additionally, we identified post-translational modifications, which according to the literature are associated with the development of colorectal cancer, including kininogen 1 protein (T327-p), alpha-2-HS-glycoprotein (S138-p) and newly identified PTMs, i.e., vitamin D-binding protein (K75-ac and K370-ac) and plasma protease C1 inhibitor (Y294-p), which may also contribute and negatively impact on CRC progression. **Conclusions:** The contribution of cytokines and proteins of the extracellular matrix is the most significant factor in CRC development in the early stages. This can be concluded since tumor growth is tightly associated with chronic aseptic inflammation and concatenated malignancy related to loss of extracellular matrix stability. Due attention should be paid to Apolipoprotein E (APOE), Apolipoprotein C1 (APOC1), and Apolipoprotein B-100 (APOB) because of their impact on the malfunction of DNA repair and their capability to regulate mTOR and PI3K pathways. The contribution of the observed PTMs is still equivocal, but a significant decrease in the likelihood between modified and native proteins was not detected confidently.

## 1. Introduction

According to the WHO, colorectal cancer (CRC) takes third place among the most common cancers and was the second most common cause of cancer deaths in 2018 worldwide (WHO, 2017) [1]. Typically, CRC is asymptomatic in the early stages, therefore resulting in diagnosis only in the late stages of disease [2]. There are a limited number of approaches for CRC diagnosis, of which colonoscopy and biopsy are the most widely employed. Screening for tumor markers such as cancer-embryonic antigen (CEA) and carbohydrate antigen CA 19-9 is considered to be an auxiliary and non-specific method of diagnosis. Increases in their levels in the blood are weakly correlated with tumor burden, yielding many false-positive and false-negative results. However, early diagnosis of cancer may increase the surveillance of patients being cured, thus raising the success rate to 90%. In this respect, the observation of tumor-specific biomarkers applicable to the early stages of the disease is causing growing interest in biomedical research [2]. Recently, several significantly altered markers relevant to CRC progression have been announced. Although most of these are on track, the majority are still kept under strict and massive revision. Substantial attention is paid to relative abundance of carcinoembryonic antigen (CEA, sensitivity 50–80%, specificity above 80%) in combination with serum amyloid A (SAA) (sensitivity of 100%, specificity 83–100%) and also carbohydrate antigens CA 19-9 (sensitivity of 47.8%, specificity 90.1%), CA 125 (sensitivity of 57.1%, specificity 92% among CEA-negative patients), CA 72-4 (sensitivity of 50%, specificity 86%), and protein CYFRA21-1 (sensitivity up to 18.2% and 42.3% in stages A and B according to the modified Dukes’ classifications) [3,4,5,6,7,8,9,10]. Nevertheless, the specificity for CRC diagnosis is questionable, since their presence and alterations are likewise associated with the development of squamous cell and hepatocellular carcinoma, lung, and bladder cancer [11,12,13]. Additionally, case study reports mention these proteins with regard to the pathology of the liver, musculoskeletal system, organs of vision, and pneumonia [14,15,16]. Analysis using liquid chromatography and high-resolution mass spectrometry (HPLC-MS/MS) may give feedback about post-translational modifications (PTM) of proteins sampled from patients with CRC. Currently, detailed information about 20 proteins with nearly 100 post-translational modifications (PTMs) is annotated for CRC. Despite recent advances in proteomic characterization approaches, no new serological markers for CRC have been identified that might meet clinical requirements of specificity and selectivity.

Blood plasma serves as an attractive source of candidate protein markers and specific pathologies for molecular profiling as it contains molecular components secreted by cells in diseased tissues, as well as factors involved in the development of pathophysiological processes [17]. Looking for the pattern of functionally interconnected elements instead of individual ones is a more attractive and promising venture. Proteomic profiles provide information enriched by relative abundances of the proteins surveyed in normal and pathology states and their interaction. Identification of the specific protein pattern associated with the early growth and expansion of CRC may assist in attaining risk criteria related to cancer development and uncover the molecular mechanisms underlying the treatment of a malignant epithelial tumor arising from the colonic or rectal mucosa. Currently, the primary concept vectors described in the P4-medicine strategy, namely, predictive, preventive, personalized, and participatory, include the detection of pathology-specific molecular profiles, or disease “patterns” [18].

In this work we perform proteomic profiling of immunodepleted plasma samples with support by HPLC-MS/MS and further bioinformatics analysis. This complementary approach allows listing of the protein pattern and further development of a comparative analysis of differentially altered proteins, as well as the identification of PTMs associated with the development of CRC. We revealed 80 plasma proteins specific to CRC and a set of 24 proteins with alternating abundances between CRC patients and the control group. In addition, 20 proteins with corresponding 29 modified peptides were identified among which six proteins with 12 PTMs have previously been reported as being associated with colorectal cancer.

## 2. Results and Discussion

**Figure 1:**
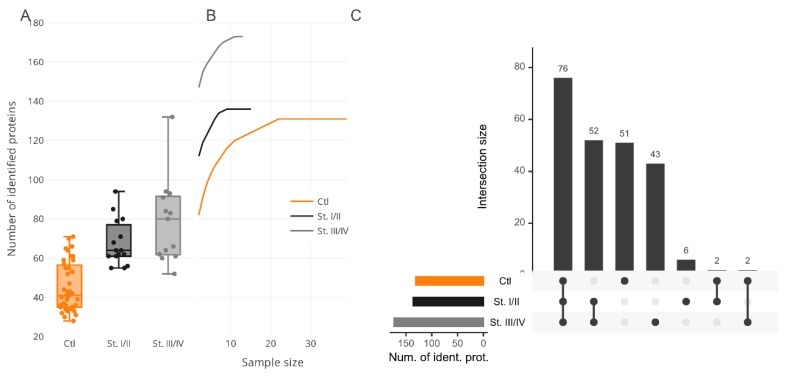
(**A**) The number of identified proteins in depleted plasma samples. Tukey box plots of a total number of identified proteins in control plasma samples and plasma samples from patients with colorectal cancer stages I/II and III/IV. (**B**) The saturation curves for protein identifications depending on the number of analyzed biosamples of cancer series (black and gray lines) and “Control” series (orange line). (**C**) The UpSet diagram shows the intersection size among cancer stages and the “Control” series.

Colorectal cancer (CRC) is a malignant epithelial tumor derived from colonic or rectal mucosa and is characterized by such clinical findings as rectal bleeding, bowel obstruction, and weight loss. Most forms of CRC develop from polyps and seldom manifest articulated symptoms. Hence, systematic screening and observation are highly recommended to prevent cancer progression in the early stages. CRC is typically observed by colonoscopy and biopsy examination and also by fecal occult blood reported by patients. Unfortunately, until the tumor has grown beyond the mucosa of the colon or rectum, the early stages of CRC development are still unrevealed or poorly available in clinical diagnostics. Hence, surgery remains the most appropriate means of CRC management and of curing CRC. Issues arise from the lack of confident and robust “alarming” markers that allow for diagnosis of CRC on time and for the taking of immediate actions to prevent tumor growth and progress. An effort to explore certain CRC-specific protein markers is a detrimental task. Thus, searching a specific symphony, or signature, of protein markers, is endorsed by professional societies as a promising venture. In this respect, we paid special attention to the determination of protein composition among surveyed proteomes that might feature CRC development in the earliest stages while the tumor has still not invaded nearby lymph nodes or adjustment tissue. Elicitation of proteins signatures, corresponding to certain pathogeneses, entails the principal question of what should these look like? Admittedly, the most appropriate way is to accomplish the search in two directions, the combination of which may give the best result and give a look at the molecular event of the disease; these two directions are qualitative categorization and quantitative characterization. In this study, we examine proteins of 28 plasma samples from patients diagnosed with CRC (in stages from I to IV); the control group comprised 41 samples from healthy volunteers with no previous history of cancer or medical records regarding chronic diseases. The median numbers of protein identifications in the plasma proteome of CRC groups are shown in Figure 1A.

To determine whether inspected bias can be the consequence of sample size unsaturation, we analyzed the cumulative effect of the group size increment on the resulting number of protein identifications (Figure 1B). In this study, CRC samples were stratified into two subgroups, namely, I–II and III–IV stages according to the classification of the TNM (Tumor, nodes, metastasis International anatomical classification system of cancers) stages (Table 1). The assayed merged subgroups showed a trend for saturation for more than 10 samples for CRC and about 20 samples for the control group, whereupon the number of proteins in the summarized proteomes was increased insignificantly. Although there are data regarding the gender-specific difference in overall survival and cancer-specific survival for CRC patients [19], unfortunately, this kind of anthropometric stratification would have been almost impossible within the frame of this study because it would have given even smaller sizes of subgroups with no possibility to review and apply any statistical significance.

The common part of the proteome shared between all the groups was determined by comparative analysis. For this purpose, the CRC group was subdivided into several fractions according to the stages of cancer progression. Hence, stages I–II included 119 proteins and stages III–IV were composed of 166 proteins; both were matched against the control group (n = 125 proteins) and returned 65 mutual protein identifications, or about 50% of matches (Figure 1C). Taking into account the results of the cumulative analysis, we assumed that proteome overrepresentation in the CRC group, as has been touched on above, is a peculiarity of the pathophysiological state suffered by patients and can be noted as one of the features specific for cancer-related groups.

Analysis of subcellular localization demonstrated that the observed proteins can be attributed to several different locations (Supplementary Materials Table S2), namely, from two localizations (28 proteins) to three (15 proteins), and even more (18 proteins), and that only nine proteins are characterized by a single localization, namely, hyaluronan-binding protein 2 (HABP2), hemoglobin subunit delta (HBD), apolipoprotein F (APOF), nucleolar protein 14 (NOP14), ficolin-3 (FCN3), coagulation factor XIII B chain (F13B), phospholipid transfer protein (PLTP), nesprin-1 (SYNE1), and DNA polymerase kappa (POLK). The majority of proteins associated with CRC are characterized as a part of secretory vesicles (12%), extracellular organelles (in particular exosomes, 31%), and general extracellular space (37%). Some proteins are related to membrane-bounded organelles or membrane-enclosed lumens (20%), and, of cause, fractions of the identified proteins are a part of lipoprotein particles, which can be attributed to protein–lipid complexes. (Supplementary Materials Table S2).

**Figure 2:**
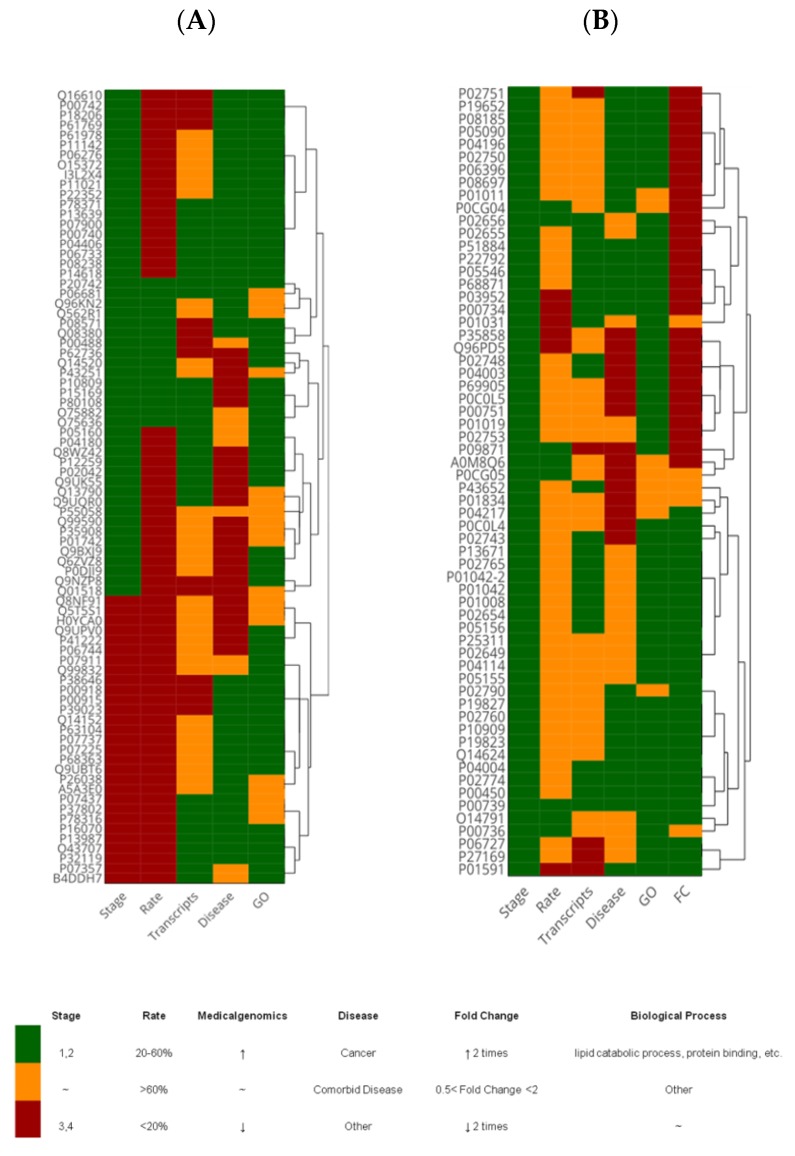
Heat map for proteins identified only in samples of patients with colorectal cancer (CRC) (**A**) and differentially expressed proteins between the two comparison groups (**B**). “Medicalgenomics” is an integrated database for BlotBase and the Library of Medical Genomics which allows for the analysis of the expression level of transcripts in the tissues of healthy volunteers and cancer patients (http://medicalgenomics.org/). “Disease” is an updated website of genre-wide association studies (https://diseases.jensenlab.org). “Rate” shows the frequency of occurrence of a protein (gene) in samples of patients with CRC and is estimated as a percentage. “Biological process” is presented in terms of GO (Gene Ontology resource) and data were obtained using the resource STRING (https://string-db.org/). “Fold change” reflects the differences in the quantitative protein content between the two series of samples, i.e., healthy volunteers and patients with CRC. “Stage” indicates the stage of development of the cancer from I to IV.

Functional annotation analysis of the CRC-specific part of the proteome revealed that the majority of proteins belong to widely interpreting processes such as metabolic reactions (GO:0044238), hemostasis (GO:0007599), positive regulation of the immune system process (GO:0002684), and response to stress (GO:0006950; Supplementary Materials Table S3). Among the CRC-specific part of the proteome, we identified 44 proteins associated with cancer development and impairments of the endocrine system including lipid metabolism (nine genes) (Figure 2); likewise, 46 proteins were characterized by increased transcript levels in large intestine biopsies and 51 proteins were indicated for the I and IIA–IIC stages of CRC (Figure 2A).

The group of CRC-specific proteins, i.e., pregnancy zone protein (PZP), complement C2, beta-Ala-His dipeptidase (CNDP1), beta-actin-like protein 2 (ACTBL2), attracting (ATRN), and ficolin-3 (Figure 2A and Supplementary Materials Table S3) have attracted noticeable attention despite the fact that they cannot be quantified. Most of the quantitatively distinguished proteins are strictly compliant with CRC pathogenesis following the transcriptome analysis of the Library of Medical Genomics (Figure 2). Over half of the significantly varied proteins are associated with cancer development or with comorbid disorders (Figure 2B) characterized by disturbance of the immune system (five proteins) and lipid metabolism (three proteins). Among the proteins related to comorbid diseases and characterized by overwhelming alterations, extraordinary attention should be paid to complement C6, ADP/ATP translocase 3 (ANT3), complement factor I (CFAI), vitronectin (VTNC), vitamin D-binding protein (GC), ceruloplasmin (CP), and haptoglobin-related protein (HPR) (Figure 2B and Supplementary Materials Table S4).

**Figure 3:**
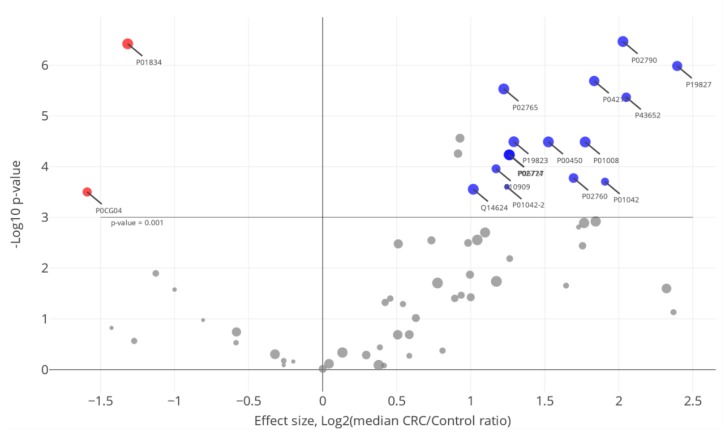
Volcano plot for comparing the relative abundance of proteins (NSAF) between the “S” series and “CC” series (stages I and II). The log2 expression ratio (biological significance) is plotted versus the −log10 of the *p* value obtained from the U-test. The upper dotted line indicates the adjusted *p* value (Bonferroni correction). Proteins with UniPtot AC are considered to have been significantly changed.

Besides group-specific proteins, the main interest was attached to the commonly identified proteins. There were 62 proteins observed with alternating abundancy levels with statistical significance of *p* < 0.001 according to the Mann-Whitney U-test between the control group and the group of patients with CRC in the I–II stages (Figure 3). Using the cut-off level of *p* < 0.05 for depletion, we found 14 proteins and two proteins with increasing fold change (FC) values (FC > 2) and decreasing (FC < 2), respectively. The size of the circle in Figure 3 indicates the co-occurrence of certain proteins in both the control and CRC (I–II stages) groups of study. The most explicitly varying proteins were involved in interconnected reactions surrounding immune response with co-occurred complement cascade activation, namely, alpha-1B-glycoprotein (A1BG), alpha-2-HS-glycoprotein (AHSG), apolipoprotein B (APOB), complement factors C4A, C6, and CFI, clusterin (CLU), haptoglobin (HP), plasma protease C1 inhibitor (SERPING1), and VTNC, and both processes were tightly linked with hemostasis and insulin-like growth factor uptake, i.e., A1BG, AHSG, APOB, CLU, immunoglobulin J chain (IGJ), inter-alpha-trypsin inhibitor heavy chain H4 (ITIH4), kininogen-1 (KNG1), antithrombin-III (SERPINC1), plasma protease C1 inhibitor (SERPING1). Due to all the reactions noted before being typically enforced through dynamic PTMs, we revealed, expectedly, an overrepresented cluster of proteins belonging to post-translation phosphorylation reactions (see also Supplementary Materials Tables S3 and S4).

Initially, the analysis was not focused on looking for certain modifications but the observed PTMs were obtained during routine qualitative analysis when several types of PTM simultaneously were selected in searching parameters for identification. This may have prevented confident identification of PTMs, and we therefore limited the number of proteins for closer examination of PTMs by sequentially sorting them according to criteria stated in the Materials and Methods section (see the “Protein Identification” section). After refinement of identification results, we revealed a fraction of 57 believable PTMs peptides corresponding to 29 proteins. Since the number of peptides/proteins was not too large, the peptides/proteins were manually curated to check the obtained identification with the raw spectra data. Following manual curation, only 25 proteins (or 42 PTM locations) survived, among which only 20 proteins with located 29 PTMs residues, survived within the proteome of interest in the CRC group of patients, unlike the control group.

**Figure 4:**
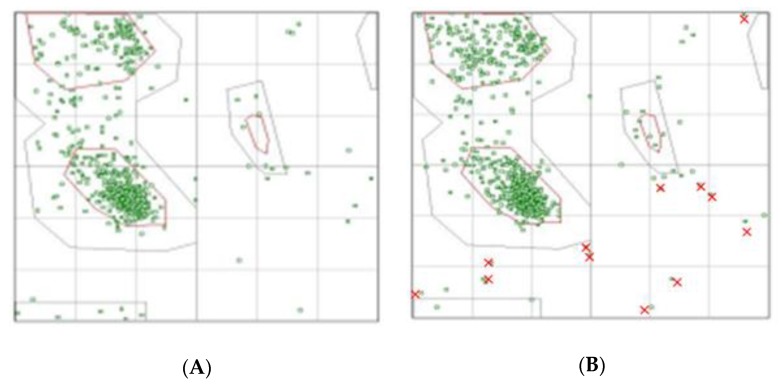
Ramachandran plot analysis for vitamin D-binding protein native structure (**A**) and modified (**B**), obtained from Protein Data Bank based on ID PDB: 1J78. Red crosses indicate amino acids that have passed into disallowed regions.

The modified residues were found in albumin, alpha-2-macroglobulin, actin, vitamin D-binding protein, and complement system components, and were exposed mostly at the terminus of α-helix and β-strands, rarely being distributed in unstructured loops linking structural domains or α-helixes and β-layers. The latter dramatically affects the spatial location of domains, and, as a consequence, the biological activity of a protein. There were 12 identified modifications which bore an association with colorectal cancer (Phosphosite, www.phosphosite.org): KNG1 (T327-p and S332-p), serum albumin (ALBU K75-ac, S82-p, K160-ac, K223-ac, K286-ac, Y287-p, and K524-ac), AHSG (S138-p and S330-p), reticulocalbin-1 (RCN1 and K86-ac), SERPING1 (Y294-p), and T-complex protein 1 subunit zeta (CCT6A K5-ac). The stereochemical qualities of the predicted models of proteins with PTMs were analyzed and the backbone conformation was evaluated by probing the Ψ/ϕ Ramachandran plots, but this did not reveal sufficient variability of the residues in disallowed regions (Figure 4, Table 2).

The backgrounds of CRC origination are still largely unknown. There are several pieces of evidence that suggest that the incidence of CRC increases with such risk factors as age, diabetes mellitus, ulcerative colitis, Crohn’s disease, and family history of colorectal cancer; there is also the hypothesis that intestinal microflora may react with bile salts and thus facilitate carcinogenic changes. Dietary contribution may also play a significant role in the genesis of CRC since refined carbohydrates and fat reduce the transit of food through the gastrointestinal tract, and, consequently, increase the exposure of mucosa to potentially dangerous carcinogenic substances [20,21]. The genetic contribution to CRC development is not so large, since up to 75% of incidences are sporadic and, for example, hereditary nonpolyposis colorectal cancer (HNPCC) accounts for 5% to 10% of CRC cases [22]. Hence, most of the genetic factors remain uncertain. However, mutations in some genes of DNA mismatch repair proteins MLH1, MSH2, and MSH6 have been linked with CRC development [23].

### 2.1. Implication of Complement Systems in CRC Development and Surveillance

In this work we obtained a wide cluster of proteins with extracellular region locations involved in immune response and complement cascade activation (GO:0030449, false discovery rate (FDR) = 1.56 × 10^−24^) following acute inflammatory reactions (GO:0002673, FDR = 1.20 × 10^−23^). The number of such immune-specific proteins varied from 22 to 51 depending on the specification of processes. The large amount of these proteins is not a bizarre event because the complement system was always considered a part of a protective mechanism against increasing the capacity of malignant cells during neoplasia [24]. However, there have been reports that the complement system can also promote tumor growth in the background of acute inflammation [25]. The root of the immune response is concluded in the ability of cancer cells to produce different antigens due to series genetic rearrangement in growing malignant cells [26]. Such tumor-associated surface antigens distinguish malignant cells from normal ones and induce an immune-mediated response. This may support further mechanisms of recognition and eradication of nascent tumors through triggering the immune responsive network [26,27].

At this point, the nascent tumor generally does not manifest clinical symptoms and it is quite difficult to obtain clinical evidence of tumor growth and progression; it is also difficult to elucidate how often the tumor is eradicated. The mechanism of the immune response against nascent tumors is widely unknown, which is one of the reasons for poor diagnosis of early cancer development. The immune response is regulated in several ways. In this work we found several well-known soluble in plasma regulators, namely, CLU, C4b-binding protein alpha chain (C4BP), and CD59 glycoprotein [24,28], and all these factors were found to be up-regulated in the CRC group in stages I and II. C4BP is probably one of the most important regulators among other regulators. It controls the classical and lectin pathways by preventing assembly with complement C3, and, consequently, inactivating complement C4b [29]. The abundance of C4BP was increased by up to 1.59 folds change in CRC patients and the lectin pathway was specifically found to be enhanced in patients with CRC compared to healthy subjects.

CLU was also observed in increasing abundance (FC = 2.2), but this factor acts on the terminal stage of complement activation. Additionally, it is known that CLU can regulate the production of pro-inflammatory cytokines such as tumor necrosis factor (TNF-α) and interleukin (IL-6) [30]. However, CLU as well as CD59 (the CRC-specific molecule in our study) both terminate the polymerization of membrane attack complex (MAC), thus preventing exceeded cell lysis and maturity of inflammation. Concerning CD59, it should be noted that this membrane-bound complement regulatory protein is one of the antigens typically expressed on the surface of cancer cells, whatever their origination. Its increasing concentration was found to be associated with resistance to cell lysis, hence enhancing and promoting tumor growth and expansion [31,32]. However, the expression of CD antigens is equivocally interpreted: for example, the expression of CD55 is associated with colorectal carcinoma [33], but the loss of CD55 is associated with breast cancer [34,35].

Among significantly alternating complement factors, C1r and C1s should be mentioned as being down-regulated in CRC groups. Because their regulation is directly accomplished through the activity of the C1 inhibitor [36], we assumed that its significantly increasing concentration would have to be observed and expected in CRC patients due to C1r and C1s down-regulation (FC = 0.41 and FC = 0.57, respectively). However, the C1 inhibitor was not detected or identified in our research. The explanation of this phenomenon may be highlighted in the significantly increased abundance of KNG1 (FC = 3.24) and, apparently, plasma kallikrein (KLKB1, FC = 1.33) which are quantitatively linked with C1 inhibitor deficiency [37]. Apart from the coagulation and complement systems, the latter (C1 inhibitor) is a known regulator of the kinin–kallikrein system. The deficiency in the C1 inhibitor is also reflected in the abundantly elevated level of alpha-2-plasmin inhibitor (SERPINF2, FC = 1.71) in CRC patients.

### 2.2. Relationship between Inter-α-Trypsin Inhibitor Heavy Chains and CRC Development

The inter-α-trypsin inhibitor family comprises serine protease proteins compiled of light and one or two heavy chains [38]. The light chain is represented by the only type and is encoded by the AMBP protein, while the heavy chains (ITIH family) exist in five different homolog forms and are encoded by different genes. These protein families were specifically underlined in view of their involvement in the re-modeling of extracellular architecture which is crucial for the malignancy process and cell migration [39,40]. In turn, AMBP is a precursor of α1-microglobulin (A1M) which is capable of accomplishing peculiar independent functions in the regulation of hemostasis, protection from oxidative stress, and negative regulation of immune response through interaction with the lymphocytic surface CD79a antigen [41,42].

Recently, the involvement of ITIH in different pathophysiological processes, including inflammation and carcinogenesis, has been discovered and extensively elucidated [43,44]. These proteins are positively or negatively regulated depending on the conditions, but there is certain strong evidence that all ITIH family members play a significant role in cell malignant processes and tumor growth [39,45]. Up until now, there is no systematic approach to evaluating differential abundances of ITIH family proteins in cancer development due to limited sensitivity and specificity, and thus the modality for early detection of CRC is also limited [46].

Apart from the ITIH1 and AMBP, which are generally upregulated [39,45], each member of the ITIH family depicts differential expression levels in various tumors [45]. These proteins (ITIH1 and AMBP) in our research also exhibited increased plasma levels in CRC in stages I and II and displayed FC values of 5.05 and 2.94, respectively (Supplementary Materials Table S4). There was no statistically significant difference in the plasma level of ITIHs between various subgroups of CRC patients stratified by clinical characteristics [45]. However, different expression levels were observed for ITIH3 and ITIH4, which were down- and up-regulated, respectively, in CRC patients. These results particularly agreed with data obtained in our research when we demonstrated a considerably increased level of ITIH4 (FC = 2.02, Supplementary Materials Table S4) in patients in the I and II stages. Nevertheless, another family member, ITIH2, is typically exhausted or displays significant down-regulation in CRC pathology (up to 61% of tumor cases, [47]), which is at odds with the obtained results in our study on positive regulation of ITIH2 (FC = 2.18, Supplementary Materials Table S4). Previously, a strong link between the expression levels of ITIH2 and estrogen levels has been demonstrated, since ITIH2 contains an estrogen-binding domain which might be critical for metastasis and tumor growth because estrogen has a profound effect on extracellular matrix integrity [48]. However, due to the fact that our results do not completely agree with earlier established reports, we are reluctant to make a final conclusion in relation to ITIH2 levels in CRC patients. Nevertheless, the role of ITIH proteins in carcinogenesis through proper stabilization of the extracellular matrix, hence preventing tumor growth, is noticeably fundamental.

### 2.3. Repercussion of Proteins with Widely Proposed and Yet to Be Elucidated Roles

As far-fetched as it seems, proteins largely encompassing molecular functions leave only a very narrow margin for valuable prognosis. We considered a set of such proteins, namely, alpha-1-acid glycoprotein 2 (ORM2), A1BG, HP, hemoglobin subunits HBA1 and HBB, and leucine-rich alpha-2-glycoprotein (LRG1) (Supplementary Materials 4) where the principal position belongs to LRG1 because it forms direct functional connections with all other members (protein-protein interaction PPI = 3.64 × 10^−14^, average local clustering coefficient 0.806) aside from HBA1. Almost all these proteins are involved in the biological process of stress response (GO:0006950) and vesicle-mediated transport (GO:0016192), with regular relevance in relation to cancer of independent etiology [49,50].

The LRG1 is a multipotent protein involved in positive regulation of angiogenesis, transforming growth factor receptor (TGF-βR) pathway signaling, and neutrophils degranulation. It is believed to be implicated in cell surveillance and regulation of apoptosis by down-regulation of cyclin D1 and apoptosis regulator Bcl-2 [49,51]. Although these findings may indicate that LRG1 can be one of the key points in promoting cell proliferation and inhibiting cell apoptosis, and given that up-regulation has been reported in several types of carcinoma, its exact role in CRC development and tumorigenesis remains poorly understood.

The relationship between LRG1, ORM2, and A1BG was established through investigating the depletion of LRG1 and affected runt-related transcription factors (RUNX) [52]. Insofar as RUNX factors are tightly associated with TGF-β signaling pathway activation, it was supposed that an increased level of up-stream proteins (LRG1, ORM2, and A1BG) markedly enhances cell proliferation and promotes tumor growth in the early stages of cancer progression [53]. Other research has reported that LRG1 is associated with CRC via regulatory induction of HIF-1α and modulating Smad 1/5/8, thus contributing to cell migration, invasion ability, and angiogenesis [54]. All the aforementioned studies have reported significantly higher plasma levels of LRG1 in CRC patients in comparison to healthy volunteers, with complete compliance found within the results obtained in our research when the LRG1 level increased to FC = 1.81 and ORM2 was elevated to FC = 1.26 (Supplementary Materials 4). Expression levels and protein abundance of these serological markers are closely correlated and are thus extensively utilized as favorable prognostic markers in patients with CRC already undergoing stage II of cancer [55].

Functional annotation of A1BG is yet poorly understood, although it is overexpressed in several types of cancer, including CRC [56,57], and is ubiquitously expressed in many tissues under normal conditions. While studying CRC, A1BG has been observed to be abundant vesicles, suggesting its role in modulation of the immune response during cancer development [58]. The A1BG protein shows significant homology with immunoglobulin family proteins. Increasing abundance of A1BG is typically co-occurred together with the C3 complement factor, assuming that this protein might be involved in immune-mediated inflammatory reaction [59]. In this work we detected a duet of elevated levels of A1BG (FC = 3.56) concatenated with raising the level of different complement factors, as has been touched on before. This leads to the suggestion that patients with CRC in stages I and II manifest immune response through a drastically activated complement cascade, supported by overexpression of A1BG.

It is probably that rather less valuable and specific markers are HP, HBB, and HBA1. The main role of HP is iron transport and its capability to bind with hemoglobin. The concentration of HP with HBB and HBA1 is strongly reversely correlated, and it has been reported that iron is a favorite for tumor growth, being primarily accumulated in cancer cells [60,61,62]. In this respect, we observed, expectedly, a raising of the level of HP (FC = 3.29) and HBA1 (FC = 1.70) in plasma samples of CRC patients in stages I–II whereas, oddly, the abundance of HBA1 was nearly that of the control level (FC = 1.04). Despite the detection of these proteins as seemingly prominent, their level alteration is accompanied by many pathologies, including anemia, thrombocytopenia, diabetes mellitus, liver, and kidney diseases, and the prognostic value of HP, HBB, and HBA1 is questionable.

### 2.4. The Prognostic Significance of Lipid Metabolism

Apolipoproteins are a large family of proteins mainly involved in lipid particle remodeling, lipid transport, binding, and clearance. Changing their levels covers a wide variety of pathologies [63]. Some of them, like apolipoproteins APOE, APOC1, and APOA1, are associated with aggressive behavior of tumor growth [64]. In this work we determined a series of apolipoproteins (APOA, APOB, APOC1, APOC2, APOC3, APOD, APOE, and APOL1; see Supplementary Materials 4) which in one way or another are implicated in the development and progression of cancer [65]. Apart from APOC2 and APOC3, these apolipoproteins displayed meaningful up-regulation in the studied population. The most elevated levels were detected for APOE (FC = 3.02) and APOB (FC = 2.55), and both have been reported to be increased in CRC patients [64,65]. A recent study showed that overexpression of APOE is associated with the advanced stage of tumor growth and enhances the risk of metastasis and invasion [65]. A possible reason for this is linked with the key role of APOE in cell proliferation and DNA synthesis; hence, impairments in one of these molecular functions may follow to tumorigenesis [66]. On the other hand, APOE is one of the regulators for the PI3K/Akt/mTOR pathway, which plays a crucial role in cell migration and proliferation, and, consequently, fosters tumor progression by enhancing cell polarity [67], hence inhibiting apoptosis in CRC cells. The value of APOE in CRC has been reported as its being a prognostic rather than predictive marker to elucidate progression between stages I and II caused by possible interaction of APOE expression levels and DNA repair functionality [64].

Although APOC1 contributes to poor prognosis in CRC, its increased levels are well established in this pathology [68], which is in agreement with our results (FC = 2.00). Another apolipoprotein (APOD) manifests contradictory and highly unusual behavior. The inverse correlation of APOD with tumor progression, particularly in the CRC case, is a known phenomenon [69,70]. According to these studies, our finding of FC = 1.49 assumingly corresponds to the early stages (I and II) of cancer development. Nevertheless, this outstanding phenomenon creates a paradox situation, since APOD expression is boosted by increased reactive oxygen species (ROS) concentration and there is a lot of growing evidence which suggests that cancer development involves ROS generation following oxidative stress [70]. Still, the APOD level can serve as a marker of early stages of cancer rather than tumor progression after initiation.

The circulating level of APOA1 in plasma is expected to be decreased in CRC patients, as has been repeatedly demonstrated in numerous works [1,65]. This suggestion is based on the obesity-associated risk of cancer development [65]. It is also favorable to measure the ratio of APOB/APOA1 as a more confident sign of CRC because APOB alone does not significantly correlate with tumor growth [71]. While the abundance of APOB is likely increased, the level of APOA1 is typically decreased during CRC. In particular, we obtained agreed upon results for APOB (FC = 2.25), but the level of APOA1 was also up-regulated in the studied CRC cohort, giving FC = 2.62. The obtained results partially contradict our expectations; however, this might be caused by the fact that the apolipoproteins family is associated with overall risk of cancer development rather than a certain type of tumor pathogenesis. Another side of the APOB prognostic value can be seen in its post-translational modification, since glycated APOB is associated with dysplastic and neoplastic tissue in CRC.

### 2.5. Post-R\Translational Modifications of Proteins

Post-translational modifications play a pivotal role in the negative functionality of proteins. They are due to the covalent bonding of chemical groups. Some modifications may occur when a protein is released from ribosomes. We compared non-modified protein structures with proteins containing post-translation modifications or modified proteins.

There is a slight drop in the Z-score between the native and modified proteins of three proteins (Table 2): complement C4-A (ID PDB: 5JPN), alpha-2-macroglobulin (ID PDB: 4ACQ) and vitamin D binding protein (ID PDB: 1J78). However, retinol-binding protein 4 (ID PDB: 1BRP) shows a dramatic increase in Z-score in comparison to the values of the Z-score of other modified proteins.

The score expressing how well the backbone conformations of residues correspond to the known allowed areas in the Ramachandran plot is within expected ranges for well-refined structures without ligands. As demonstrated in Table 2, most of the identified modifications do not significantly affect the change in Z-score of the protein structures. The Ramachandran plot provides an easy way to view the distribution of torsion angles in a protein structure. It also provides an overview of excluded regions that show which rotations of the polypeptide are not allowed due to steric hindrance (collisions between atoms). The Ramachandran plot of a particular protein may also serve as an important indicator of the quality of its three-dimensional structures.

The protein modifications identified in our PTM study, as a rule, do not lead to significant changes in values of score and Z-score. Only for one vitamin D-binding protein which contains two PTMs (K75-ac and K370-ac) are several amino acids in the disallowed regions visible on the Ramachadran plot (Figure 4). However, in general, this did not lead to a significant decrease in the likelihood of such a structure.

All this allows us to conclude is that the identified 29 PTM for 20 proteins are also specific to blood plasma samples obtained from patients with colorectal cancer.

## 3. Materials and Methods

### 3.1. Demography

The materials chosen for this study consisted of plasma samples of venous blood obtained after an overnight fasting process. We studied 41 control blood plasma samples from healthy volunteers and 28 samples from patients with colorectal cancer provided by the laboratory of clinical biochemistry of the N. N. Blokhin Russian Cancer Research Centre (Table 1, Supplementary Material 1). Written consents were obtained from the patients for participation in the study and the use of their biological material.

Blood was collected in pre-chilled tubes with ethylenediaminetetraacetic acid (EDTA), quickly mixed, and centrifuged at 4 °C and 1500 rpm for 10 min; blood plasma samples were immediately collected and frozen. After centrifugation, the supernatant in the tubes was carefully collected with an automated pipette and placed in nine cryovials of 2 mL [72].

This study was approved by the independent local research ethics committee of the Institute of Urology and Reproductive Health (Sechenov University) (protocol no. 10-18 of November 7, 2018). Written informed consent was obtained from the patients and healthy volunteers authorizing their participation in the study and the use of their biological material. All the samples were deactivated before their use in the study to provide biological safety.

### 3.2. Sample Preparation for MS Analysis

We followed the methods of A. L. Kaysheva et al. 2019 [73]. Blood plasma at a volume of 40 µL was brought to a final volume of 500 µL by adding a solution of 0.1% deoxycholic acid sodium salt, 6% acetonitrile, and 75 mM triethylammonium bicarbonate, pH 8.5.

Enzymatic cleavage of proteins was performed using a specific trypsin protease. The protein solution was added to with modified (acetylated at primary amino groups of lysine) trypsin at an enzyme-to-substrate ratio of 1:50. After this, a second aliquot of trypsin was added at a ratio of 1:100 and incubated at 37 °C, which was continued for an additional 12 h [73]. The sample preparation protocol is presented in detail in the PRIDE Project PXD015163.

### 3.3. Mass Spectrometry Protein Registration

Analysis of the prepared depleted plasma samples was conducted using a high-resolution mass spectrometer Q Exactive-HF (Thermo Scientific, Waltham, MA, USA) equipped with an adapted for nano-flow NSI ionization source (Thermo Scientific, Waltham, MA, USA). The instrument was operated in positive ionization mode. Precursor ions were surveyed within the range of 420–1250 *m*/*z* at a resolution of R = 60 K and accumulated for a maximum integration time of 15 ms, or until an acquisition gain control (AGC) of 3e6 ion was reached. The selection of a certain *m*/*z* range for detection was based on the requirements of the HUPO Guideline (bullet 9, version 3.0.0, released October 15, 2019; [74]) for a minimal length of the detected peptide for consideration and justification of PE1 proteins (according to the Uniprot KB Classification).

Top 20 ions with charge states of z = 2+...4+ were triggered for activation in high-energy collision-induced dissociation (HCD) with nitrogen (N_2_) gas at 27% normalized collision energy (normalized to *m*/*z* = 524, z = 2+) stepped within ±20% of the normalization value. Fragment ions were detected at a resolution of R = 15K and accumulated for a maximum integration time of 85 ms, or until AGC reached 1 × 10^5^ ions.

Peptides were separated on an Ultimate 3000 Nano-flow UPLC system (Thermo Scientific, Waltham, MA, USA). Samples in amounts of 2 μg were loaded onto the enrichment column Acclaim Pepmap^®^ (5 mm × 0.3 mm, 300 A pore size, 5 µm particle size) for 4 min at a flow rate of 20 μL/min in a mobile phase comprising a water solution of 2.5% acetonitrile, 0.1% formic acid, and 0.03% acetic acid. After loading, peptides were separated on an analytical column Acclaim Pepmap^®^ (75 µm × 150 mm, 1.8 µm particle size, 60 A pore size) at a flow rate of 0.3 μL/min using a gradient of mobile phases A (water) and B (acetonitrile), with both supplied with 0.1% formic acid and 0.03% acetic acid. The gradient of elution started from 2.5% B for 3 min and was raised to 12% B for the next 15 min, then to 37% B for the next 27 min, and to 50% for the next 3 min. The gradient was rapidly increased to 90% B for 2 min and was kept for 8 min at a flow rate of 0.45 μL/min. During this time segment, the mass spectrometer was switched to idle scan mode. After washing the column in a high concentration of non-polar solvent, the system of both columns (enrichment and analytical) was equilibrated under the initial gradient conditions (2.5% B) for 13 min at a flow rate of 0.3 μL/min before starting the next sample. The recorded raw data were converted to peak lists for search engines and deposited to the ProteomeXchange Consortium via the PRIDE partner repository with the dataset identifier PXD015163.

### 3.4. Protein Identification and Criteria Selection for Post-Translational Modifications.

Peak lists obtained from MS/MS spectra were identified using OMSSA version 2.1.9. The search was conducted using SearchGUI version 3.2.20. Protein identification was conducted against a concatenated target/decoy version of the Homo sapiens complement of the UniProtKB (88703 (target) sequences). The decoy sequences were created by reversing the target sequences in SearchGUI. The identification settings were as follows: trypsin specific with a maximum of two missed cleavages, 10.0 ppm as MS1, and 0.05 Da as MS2 tolerances. Variable modifications were oxidation of Methionine. The following were selected as variable modifications: acetyl (protein N-term), acetyl (K), phospho (S), phospho (T), phospho (Y), and GlyGly (K). Peptides and proteins were inferred from the spectrum identification results using PeptideShaker version 1.16.11. Peptide spectrum matches (PSMs), peptides, and proteins were validated at a 1.0% FDR estimated using the decoy hit distribution.

PTMs were observed during routine qualitative analysis according to the selected types of variable modifications. Since several types of PTMs simultaneously were selected for the searching, the returning of false-positive results was highly probable. To refine the search results, we selected only those that fit a combination of the following requirements: (a) high confidence (at least 98%) of peptide identification, (b) sufficient coverage of peptide sequence (at least 80%) (inclusion of fragment ions bearing PTM residues was obligatory), and (c) sufficient D-score for PTM probability (at least 10 units). Following engine-supported pre-selection of the identified PTMs, manual curation within the raw spectra data was accomplished.

### 3.5. Data Analysis

For statistical analysis (comparison of plasma proteome of normal and CRC cases) only proteins with two or more identified peptides were chosen (the search output from the comparison experiments was filtered to > 1 peptides). Then, normalized spectral abundance factor (NSAF) values for each protein were summated. The MARS (multiple affinity removal system) depleted proteins were excluded from the comparison lists. Statistical analyses were carried out using in-house scripts written in R [75]. The UpSet plot was generated using the UpSetR R library [76]. Volcano plots and box plots were generated using the Plotly package [77]. Cumulative curves for identified proteins and heatmaps were generated using in-house scripts written in R.

### 3.6. Analysis of Post-Translational Protein Modifications

We used the annotated 3D protein structures available in the protein data bank (PDB, https://www.rcsb.org/). In the protein structures we were able to reveal post-translational modifications with the aid of Vienna-PTM 2.0 (http://vienna-ptm.univie.ac.at/) to explore protein PTMs using molecular dynamics (MD) simulations. We modified protein PDB files with one or more supported PTMs and obtained the force field parameters (GROMOS 45A3, 54A7, and 54A8) and the input files needed to perform MD simulations of the modified proteins using the GROMACS package [78].

Validation of the modeled structure was carried out using the PROCHECK server (http://www.ebi.ac.uk/thornton-srv/software/PROCHECK/) for stereo-chemical analysis of dihedral angles in modeled protein structures. PROCHECK was used to analyze the overall residue by residue/structural geometry as determined by Ramachandran plot. We calculated the Z-score for native and modified protein structures. The Z-score indicates overall model quality and measures the deviation of the total energy of the structures with regard to the energy distribution derived from random conformations [78,79]. Hence, the Z-score is used to recognize the native folds from another alternative. The Z-score varies tremendously from protein to protein.

The formula used to calculate the protein Z score is

Zmisfolds = <E>misfolds – Enative ÷ σmisfolds(1)

where Enative is the energy of the native structure of a protein, <E>misfits is the average energy of a group of misguided units and σmisfolds is the standard deviation of the energy in this misfolded unit.

The predicted model of proteins from PDB DB (non-modified structures) and Vienna-PTM 2.0 (modified structures) were validated using qualitative model energy analysis (QMEAN) and PROCHECK. The QMEAN Z-score was another tool used for quality assessment where it analyzed the degree of nativeness of the predicted 3D structure of a protein. The QMEAN score imitated the predicted global model reliability, ranging 0–1 [78]. PROCHECK provided a detailed analysis of the stereochemistry quality of the 3D protein structure. The Ramachandran plot with Phi/Psi was provided by PROCHECK to validate the backbone structure of a protein.

## 4. Conclusions

Only a couple of decades ago did medicine and biology know almost nothing about the molecular mechanisms of tumorigenesis. Recently, proteomic approaches have supported the moving beyond of traditional opportunities in the clinical diagnosis of cancer. Despite a lot of information and details about the fundamentals of the malignancy process and cancer development being reported annually, still, we know little, and our understanding of the very early events of cancer is limited.

In the present study we aspired to elucidate the proteomic signature of CRC development based on quantitative and qualitative assessments. We paid particular attention to patients with initial (I and II) stages of cancer development. The recovered arrangement of examined proteomes highlighted known mechanisms of tumorigenesis in CRC.

It should be emphasized that the majority of proteins with significant alterations were related to immune-mediated response, including complement activation. This finding is in agreement with the well-established phenomenon of immune-mediated response caused by different antigens expressed on the surface of malignant cells. In this way, recognition and eradication of the nascent tumors happen and early stages may occur asymptotically. This can be one of many other reasons for the poor diagnosis of cancer in its initial stages.

Like many other pathologies, CRC is, admittedly, a complex disease, and therefore a burden of self-management tasks and responsibility for curation of patients with nascent tumors is a matter of special care. There is no single and strong protein marker for CRC detection in the initial stages of tumor growth, as has been shown in the present study, but rather, their proper combination may help to distinguish undesirable signs and evaluate the risk for patients.

In summary, we suggest that C4BP and CLU may hold the primary position among other cytokines due to their prognostic value, specifically in patients in stages I-II of CRC. The burden of functional connection of CD59 with CLU may enhance its value due to association with cell lysis resistance and, consequently, the encouraging of tumor growth, but an expression of different CD antigens and their implication in tumor expansion remains uncertain. Since tumor progression is tightly associated with re-arrangement of the extracellular matrix, employment of AMBP, ITIH1, and ITIH4 may also support the net benefit for early detection of CRC and designation of probable cell migration. However, results of ITIH2 measurement contradict the previously reported data [47,48], which makes early recognition of CRC less confident and specific (Supplementary Materials Table S5).

From our point of view, the panel can also be complemented by HP as an iron carrier favorable for tumor growth and by A1BG due to its strong correlation with cytokines responsible for inflammation, specifically in patients in stages I and II. The multipurpose activity of LRG1 linked with TGF-β and HIF-1α mediated signaling easily permits the subjection of this protein to being a valuable auxiliary marker of the malignancy process.

Finally, APOB, APOE, and APOC1 (particularly, APOD as a marker of increased ROS production) at least may contribute to the detection of CRC in its early development as a constellation of supportive markers, enhancing the net value of the aforementioned stronger markers. However, due to their ubiquitous activities and involvement in a wide variety of pathologies, the abundantly represented apolipoproteins may place their prognostic value at risk.

We also processed the input of post-translational modifications for some proteins under consideration but did not observe a significant decrease in their likelihood caused by PTM moieties. Most of the identified post-translational modifications were identified as being associated with the numerous types of oncological disorders, whereas some newly identified ones might be involved in the pathogenesis of CRC.

## 5. Limitations

The limitations of this study were the small size of cohorts under consideration and the insufficient size of clinically stratified subgroups, which may have caused a gross variance of some parameters. This may weaken the application and significance of the conclusion. The age factor plays a particular role in the statistics of CRC incidences. According to information given by the Colorectal Cancer Alliance, the median age for diagnosis of colon cancer is 68 years in men and 72 years in women, while for rectal cancer it is 63 years for both genders. Although the rate of incidence in people younger than 50 years old has increased to 11% (in 2013), variation in the median age between the group of patients under consideration and the control group in the present study may also affect the explanation of the final results. It should also be noted that there are several identified markers which provide the opportunity to distinguish colorectal cancer generally and in its early stages (I–II), and the majority of the observed markers are in compliance with previously reported findings; however, due care must be taken for their consideration.

## Figures and Tables

**Table 1:** Data of colon cancer patients.

Participants	Healthy Volunteers	All Patients	Clinical Stage	
			I–II	III–IV
Number of samples	41	28	15	13
Age, mean	47.9	59.8***	59.7	58.4
Men	20	14	8	6

Note: Statistically significant values are marked by an asterisk. *** *p* < 0.001, Student *t*-test.

**Table 2:** The Ramachandran plot data and the G-factor and Z-score of the proteins in question. From the Ramachandran data in the table we used the service PROCHECK, based on the analysis of structures of resolution of at least 2.0 Å. A good quality model would be expected to have over 90% in the most favored region. Similarly, we also found the G-factor score of the proteins. The G-factor provides a measure of how unusual or out of the ordinary a property is. A G-factor value which is below −0.5 is considered to be unusual; a value below −1.0 is considered to be highly unusual. The analyzed protein structures were characterized by a G-factor of more than −1.0.

Protein Name	Gene Name	ID PDB*	PTM	Residues in Most Favored Regions (%)		Residues in Additional Allowed Regions (%)		Residues in Disallowed Regions (%)		∆Score** (Z-Score)
				Without PTM	With PTM	Without PTM	With PTM	Without PTM	With PTM	
Complement C4-A	C4A	5JPN	K1594-ac	90.7	90.7	8.5	8.6	0.4	0.5	0.25 (0.246)
Alpha-2-macroglobulin	A2MG	4ACQ	K608-ac	78.7	78.5	18.0	18.1	0.7	0.9	0.23 (0.4)
Serum albumin	ALBU	1AO6	K75-ac, K286-ac, Y287-p	89.1	88.7	10.8	10.9	0	0.3	0.13 (0.013±0.027)
Vitamin D-binding protein	VTDB	1J78	K370-ac	89.6	90	10	8.7	0	0.1	0.25 (1.04)
Plasma protease C1 inhibitor	SerpinG1	5DU3	Y294-p	82	82.1	16.3	16.0	0	0.6	0.24 (−0.11)
Apolipoprotein A-IV	ApoA4	3S84	S174-p	97.2	97	2.8	2.8	0	0.2	0.58 (−0.185)
X-ray repair cross-complementing protein 6	XRCC6	3K77	S76-p	86.3	86.4	13.1	13.0	0	0	0.23 (0.16)
Alpha-1-acid glycoprotein 2	A1AG2	3APU	K138-ac	94	94	6	6	0	0	0.23 (0.25)

ID PDB*—the 4-character unique identifier of every entry in the Protein Data Bank; ∆Score**—difference in G-factor score between unmodified and modified protein structures.

## Data Availability

The mass spectrometry proteomics data are available via the ProteomeXchange repository with the dataset identifier PXD015163 (https://www.ebi.ac.uk/pride/archive/projects/PXD015163).

## Supplementary Materials

The following are available online at https://www.mdpi.com/1420-3049/25/3/619/s1, Supplementary material includes data of colon cancer patients, the list of proteins that are specific to the biological samples from patients with colorectal cancer in the first and second stages, the list of proteins, and the level which differs between the series.
